# Predicting the COVID-19 vaccine receive intention based on the theory of reasoned action in the south of Iran

**DOI:** 10.1186/s12889-022-12517-1

**Published:** 2022-02-04

**Authors:** Roghayeh Ezati Rad, Kobra Kahnouji, Shokrollah Mohseni, Nahid Shahabi, Fatemeh Noruziyan, Hossein Farshidi, Mahmood Hosseinpoor, Saeed Kashani, Hesamaddin Kamalzadeh Takhti, Mehdi Hassani Azad, Teamur Aghamolaei

**Affiliations:** 1 Student Research Committee, Faculty of Health, Hormozgan University of Medical Sciences, Bandar Abbas, Iran; 2 Social Determinants in Health Promotion Research Center, Hormozgan Health Institute, Hormozgan University of Medical Sciences, Bandar Abbas, Iran; 3 Cardiovascular Research Center, Hormozgan University of Medical Sciences, Bandar Abbas, Iran; 4 Anesthesiology, Critical Care and Pain Management Research Center Hormozgan University of Medical Sciences, Bandar Abbas, Iran; 5 Department of Community Medicine, School of Medicine, Hormozgan University of Medical Sciences, Bandar Abbas, Iran; 6 Infectious and Tropical Diseases Research Center, Hormozgan Health Institute, Hormozgan University of Medical Sciences, Bandar Abbas, Iran

**Keywords:** Theory of reasoned action, Coronavirus, COVID-19 vaccine, Iran

## Abstract

**Background:**

Vaccination against Covid 19 disease was based on rational practice theory.

One of the most effective methods to control the COVID-19 pandemic is extensive vaccination coverage in the shortest time. The relevant beliefs and predictors of COVID-19 vaccine and the barriers to and facilitators of receiving COVID-19 vaccine should be identified. Individuals’ intention to receive COVID-19 and the effective factors are of an utmost importance. This study aimed to predict intention to receive COVID-19 vaccine in the South of Iran.

**Methods:**

This cross-sectional study was performed over a period of 2 months (May 2021 to July 2021) in 4 southern provinces in Iran. The study population of this study included people over 18 years of age who did not receive the COVID-19 vaccine. The online questionnaire was used to collect data. We recruited participants through a self-selection sampling method and posted the online survey link. The questionnaire had two parts: demographic information and Theory of Reasoned Action (TRA) questions. All statistical calculations and hypotheses tests were performed using SPSS21 and Amos21 software and the significance level was considered 0.05.

**Results:**

A total number of 2556 people participated in this study with a mean age of 37.76 (10.7) of years (Age Range = 18–75). The findings showed that attitudes and subjective norms and the use of social media predict the intention to receive COVID-19 vaccine. SEM showed that attitude (β = 0.596, *P* < 0.001), subjective norms (β = 0.265, P < 0.001) were significant predictors of vaccination intention. In this study, 78% of people were willing to receive the vaccine when they were officially allowed to.

**Conclusion:**

According to the results of the study, it is suggested to strengthen positive attitudes and subjective norms about the importance of COVID-19 vaccination as well as using social media to inform the community in order increase the intention to vaccinate COVID-19 and increase vaccine coverage.

**Supplementary Information:**

The online version contains supplementary material available at 10.1186/s12889-022-12517-1.

## Background

Respiratory infections have special importance due to their rapid and wide spread and their role in mortality of community’s members [1]. The COVID-19 pandemic has caused almost unimaginable damages to the lives, health and economy of many countries. Along with health and behavioral control measures, vaccination is the most successful method to control the COVID-19 pandemic [2].

Although vaccination is an effective method to reduce and eliminate diseases, its effect depends on the willingness of the community to receive the vaccine [3]. Vaccination against COVID-19 disease is related to factors such as the speed of vaccine production and spread, relatively new vaccine preparation techniques, and the need to continue preventive behaviors even after receiving the COVID-19 vaccine [4–6]. A systematic review of COVID-19 vaccination in 31 countries showed that global vaccine acceptance was decreasing from more than 70% in March 2020 to less than 50% in October of that year (as predicted) [7]. Recent studies have estimated that 25–50% of Americans do not intend to receive the COVID-19 vaccine after the availability of vaccine that this is a new challenge in health promotion [8]. The World Health Organization (WHO) recently declared vaccine hesitancy one of the ten threats to global health [9]. The rate of COVID-19 vaccination in Iran was 0.2% until April 27, 2021 [10]. An Iranian study reports that 65.7% of Iranians intend to be vaccinated against COVID-19 in November 2020 [11]. In this regard, understanding the psychological factors that explain the intention to vaccinate COVID-19 among Iranians, is important for officials and healthcare providers in order to increase the rate of receiving COVID-19 vaccine.

TRA which considers behavioral intention as the best predictor of behavior, is driven by two main structures. Attitudes are beliefs and feelings about some behaviors and positive or negative values that depend on the outcome of that behavior. Subjective norms include an understanding of social norms (including the belief that reference individuals approve or reject a behavior) and the individual’s motivation to comply these normative beliefs [12].

The first step in creating effective health interventions to promote COVID-19 vaccination is to identify the relevant and predictive beliefs of the COVID-19 vaccine. Prior starting the general vaccination, sufficient time should be spent to remove the vaccination concerns, and barriers and facilitators to receive COVID-19 vaccine should be identified in order to make recommendations for designing interventions aimed at maximizing public acceptance as well as designing the appropriate messages to promote COVID-19 vaccination in order to reduce the concerns of those who are already hesitant. Therefore, this study was aimed to predict the intention to vaccinate against COVID-19 based on the TRA.

## Methods

### Study design

This cross-sectional study was conducted from May 2021 to July 2021 in the South of Iran with a web-based self-administered questionnaire. The statistical population of this study included people over 18 years old living in 4 southern provinces of Iran (*Hormozgan, Kerman, Bushehr and Fars*), who had not received COVID-19 vaccine.

Hormozgan province lies in the far south of Iran. It is located in the north side of strait of Hormoz. Kerman province resides in the northern side of Hormozgan province, while Fars and Bushehr provinces are adjacent to the western side. These four southern provinces in Iran have many sociocultural features in common.

When the present research was conducted, according to Iran vaccination document, in the whole country and the above-mentioned provinces, the medical staff as well as all population over 75 years of age were being vaccinated. The mortality rate was high in these provinces due to the incomparable temperature, inadequate vaccination, recurrent religious holidays (and the resultant overcrowd). The data collection occurred at the same time as the 5th peak of the pandemic.

Data were collected using a questionnaire designed on the *Pors Line* platform, an online survey platform in Iran (https://survey.porsline.ir) and was provided to the target group through social media. The questionnaire began with an information letter about the study’s purpose, how to answer questions, and informed consent to participate in the study.

### Sampling method

Regarding to the existing limitations due to the outbreak of COVID-19 and the impossibility of distributing questionnaires in paper form, data were sent to *Hormozgan, Kerman, Fars, Bushehr* provinces (which are the southern provinces of Iran) through various social media (WhatsApp, Telegram, Linkedin), email, channels and news agencies, public relations of University of Medical Sciences, Red Crescent, Municipality and University Student Research Committee. We recruited participants through a self-selection sampling method and posted an online survey link. After publishing the questionnaire link, the people who received it were asked to complete the questionnaire (if they wished) and send it to other people they know. Finally, the participants registered their answers by clicking the submit button. To emphasize on the greater participation of individuals in the study, messages and links to participate in the study were resent as a reminder two weeks after the first submission.

Also, with the cooperation of health centers in the studied provinces, a questionnaire link was sent to all people covered by healthcare centers in villages and cities. In this study, according to the data collection method, there was no limit on the number of samples.

On the first page of the questionnaire, the purpose of the study was clearly explained and the completion of the questionnaires was completely voluntary. Inclination criteria were at least 18 years old and not receiving the COVID-19 vaccine.

### Inclusion and exclusion criteria

The inclusion criteria were: the age over 18 years, not having been vaccinated, living in cities and villages in the 4 provinces of Hormozgan, Kerman, Bushehr and Fars.

The exclusion criteria was incomplete questionnaires.

### Data collection

The data collection tool was an online questionnaire. The questionnaire was designed based on studies conducted and articles reviewed [8, 13, 14] and the validity of the questionnaire was assessed by content validity method.

To check the content validity, the questionnaire was prepared using valid sources and books and related scientific papers and the necessary proposed corrections were made qualitatively and quantitatively with the approval of 2 experts in health education and health promotion. 7 people were consulted from different socioeconomic statuses, and their comments were used to revise the questionnaire content.

In the qualitative method, experts were asked to review the tool based on the criteria of grammar, use of appropriate words, placement of items in the right place and proper scoring, and provide the necessary feedback.

The reliability of the questionnaire was reviewed and confirmed by assessing the internal correlation of variables (calculating Cronbach’s alpha coefficient). The questionnaire consisted of two parts. The first part was demographic information including age, gender, marital status, education level, employment status, underlying diseases, history of smoking, history of individual and family infection with COVID-19, history of receiving the flu vaccine and source of information on COVID 19 vaccines.

The second part of the questionnaire included the constructs of TRA. The construct of attitude towards behavior (to what extent the desired behavior is desirable, pleasant, useful or enjoyable for the person) is influenced by the construct of behavioral beliefs (beliefs of the person about the result of performing a behavior) and outcomes evaluation (the value that a person considers about the result of a behavior) [15]. The construct of behavioral beliefs consisted of 7 questions of 5-item (highly agree to highly disagree) (e.g., I believe in the efficacy and safety of existing COVID-19 vaccines). The outcomes evaluation structure also included 7 questions of 5-Likert (very good to very bad) (e.g., the efficacy and safety of COVID-19 vaccines are very good). Attitude score was obtained from the multiplication of the behavioral beliefs construct in the outcomes evaluation construct.

The construct of subjective norms (the amount of social pressure perceived by an individual to perform behavior, that is, the reflection of social effect and influence on the individual) is influenced by the construct of normative beliefs (belief in whether certain people approve or reject the behavior) [16] and the construct of motivation to comply (individuals’ motivation to comply the wishes of others and accept their expectations) [17]. The normative belief construct consisted of 6 questions 5-Likert (highly agree to highly disagree) (e.g., my family members agree to receive the COVID-19 vaccine). The construct of motivation to comply also consisted of 6 questions 5-Likert (very important to not important at all) (e.g., family members’ advice to receive the COVID-19 vaccine is very important for me) subjective norms score was obtained from the multiplication of normative beliefs construct in the motivation to comply substructure.

The behavioral intention construct also consisted of 3 questions 5-Likert (highly agree to highly disagree) (e.g., I intend to receive the vaccine if it is time for the COVID-19 vaccine). The score of COVID-19 vaccine receive intention was obtained from the mean score of 3 related questions.

**Fig. 1:**
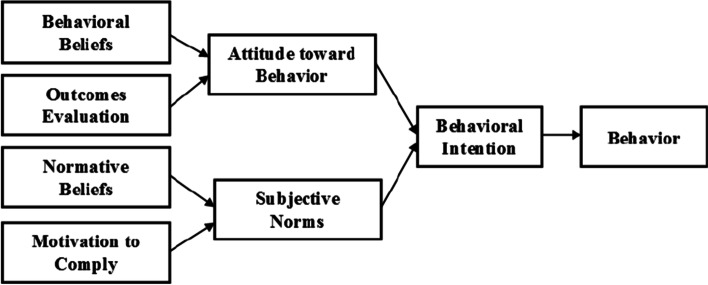
Theory of Reasoned Action – TRA (Fishbein & Ajzen, 1975)

The structure of the theory is depicted in Fig. 1.

### Statistical analysis

Frequency, percentage, mean and standard deviation indices were used to describe the data. The assumptions of parametric tests including the T-test and ANOVA were tested and confirmed initially. To test the normality of distribution, the skewness and kurtosis were tested. The skewness was divided by the skewness standard deviation to estimate Fisher’s exact test, found to range between − 1.96 and + 1.96. Thus, the normality of data was confirmed. To test the homogeneity of data, Leven’s test was used. The estimated *p*-value was over 0.05. To test the linearity of independent variables, VIF was used, which was found to be below 1.2 for all independent variables. T-test statistical tests and one-way analysis of variance were used to test hypotheses and to investigate the relationship between COVID-19 vaccine receive intention and demographic variables (age, gender, occupation, education, marital status, chronic disease, smoking, place of residence, history of receiving the flu vaccine, history of COVID-19, information sources) and multiple linear regression was used to determine the relationship between the constructs of TRA and COVID-19 vaccine receive intention. Also, the statistical technique of path analysis and structural equations modeling (SEM) were used in order to determine how the theoretical structures relate and their effect on each other, to confirm or reject the conceptual model determined for the COVID-19 vaccine receive intention.

There were no missing data in the present study. From the 3034 subjects who returned the completed questionnaires, 478 subjects (15%) stated that they had not received the coronavirus vaccine. They did not meet the inclusion criteria and were, thus, excluded from the study. The final analysis was done with a sample of 2500 subjects.

All statistical calculations and hypothesis testing were conducted using SPSS21 and Amos21 software and a significant level of hypotheses tests was considered 0.05.

### Ethical consideration

All the procedure was done in accordance with the Declaration of Helsinki. The study was approved by the ethics committee of Hormozgan University of Medical Sciences (# IR.HUMS.REC.1400.071). The ethics committee approved the online survey as well as the online consent. All participants who consented to take part in the study were assured that participation was voluntary, and that they could withdraw any time. Besides, the data were anonymized, securely stored and analyzed for publication.

## Results

The number of 3034 people participated in this study and completed and submitted the online questionnaire, but due to the fact that 478 (15.9%) questionnaires were incomplete, they were excluded from the analysis and finally 2556 questionnaires were analyzed. Accordingly, the mean age of participants in the study was 10.7 ± 37.76% and ranged from 18 to 75 years. Most participants were in the age group of 30–49 years. Demographic characteristics of participants in the study, mean score of attitude, subjective norms, and COVID-19 vaccine receive intention are presented in Table 1.

**Table 1:** Research participants’ demographic information

Demographic information	category	Frequency N (%)	Attitude Mean (SD)	Subjective Norms Mean (SD)	Intention Mean (SD)
Age group	18–29 year	567(22.2)	93.31(27.61)	83.34(26.81)	11.80(3.46)
	30-49 year	1648 (64.5)	94.31(27.52)	85.22(25.94)	12.06(3.23)
	> 50 year	341(13.3)	96.95(26.07)	90.72(25.49)	12.43(2.82)
pvalue			0.144	0.000	0.016
Gender	Male	933(36.5)	95.38(26.65)	86.38(26.43)	12.17(3.26)
	Female	1623 (63.5)	93.90(27.76)	85.05(26.00)	11.99(3.22)
pvalue			0.187	0.214	0.169
Marital status	Single	619 (24.2)	92.35(27.89)	83.73(26.52)	11.84(3.42)
	Married	1889 (73.9)	95.22(27.07)	86.08(25.90)	12.13(3.16)
	Divorced / widowed	48 (1.9)	90.56(30.67)	87.31(30.49)	11.83(3.66)
pvalue			0.047	0.137	0.132
Educational level	Non-academic	664 (26)	92.82(25.88)	86.51(26.18)	12.11(2.96)
	Associate Degree	259 (10.1)	88.89(27.60)	82.58(27.53)	11.72(3.48)
	Bachelor’s degree and higher	1633 (63.9)	95.98(27.77)	85.61(25.91)	12.08(3.31)
pvalue			0.000	0.120	0.216
Job	housekeeper	531 (20.8)	92.87(26.84)	84.85(25.85)	11.95(3.08)
	University student	275 (10.8)	95.73(28.30)	85.60(27.10)	12.17(3.40)
	Private sector employee	385 (15.1)	94.88(26.91)	86.41(25.10)	12.21(3.27)
	Public sector employee	817 (32)	95.84(27.89)	86.61(26.22)	12.07(3.32)
	Others	548 (21.4)	92.91(26.85)	83.95(26.61)	11.95(3.16)
pvalue			0.178	0.384	0.671
Chronic diseases	yes	441 (17.3)	97.29(27.72)	88.90(26.54)	12.46(2.92)
	no	2115 (82.7)	93.85(27.26)	84.83(26.03)	11.97(3.30)
pvalue			0.016	0.003	0.004
Smoking	yes	253 (9.9)	88.60(26.71)	81.09(26.83)	11.66(3.61)
	no	2303 (90.1)	95.08(27.36)	86.02(26.04)	12.10(3.19)
pvalue			0.000	0.004	0.040
Accommodation	Urban	2303 (90.1)	94.54(27.35)	85.71(25.95)	12.06(3.24)
	Rural	253 (9.9)	93.52(27.48)	83.96(27.96)	11.96(3.24)
pvalue			0.574	0.312	0.644
province	hormozgan	1451(56.8)	96.43(26.96)	87.89(25.61)	12.35(3.08)
	fars	384(15)	91.04(28.52)	81.22(27.17)	11.45(3.58)
	kerman	360(14.1)	91.00(27.38)	80.47(25.69)	11.74(3.34)
	busher	361(14.1)	93.47(27.04)	85.72(26.50)	11.84(3.26)
pvalue			0.000	0.000	0.000
Influenza vaccine	yes	508(19.9)	97.83(27.32)	87.11(25.92)	12.31(3.14)
	no	2048(80.1)	93.60(27.32)	85.14(26.21)	11.99(3.26)
pvalue			0.002	0.129	0.051
COVID-19 history	yes	1113(43.5)	94.03(27.31)	85.24(26.03)	12.04(3.27)
	no	1443(56.5)	94.97(27.44)	85.92(26.33)	12.07(3.19)
pvalue			0.392	0.514	0.864
Information Sources	social media	1215(47.5)	95.55(27.68)	85.27(25.70)	12.20(3.19)
	Radio and television	861(33.7)	93.92(25.82)	86.21(25.85)	12.03(3.05)
	Treatment staff	282(11)	96.74(28.31)	89.40(26.90)	12.25(3.35)
	scientific journals	74(2.9)	94.20(34.16)	79.88(28.86)	11.38(4.16)
	Friend and colleague	124(4.9)	82.02(24.96)	78.06(27.56)	10.78(3.77)
pvalue			0.000	0.000	0.000

**Fig. 2:**
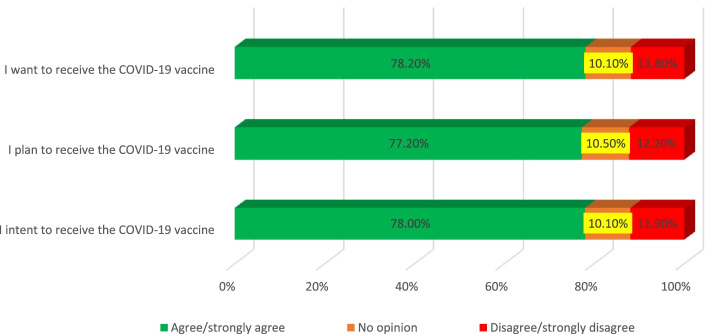
Intention of participants to receive COVID-19 vaccine

Figure 2 shows that 78% of the population intends to receive the vaccine if it is their turn to receive the COVID-19 vaccine, and 77.2% plan to receive the vaccine if it is their turn to receive the vaccine, and 78.2% want and wish to get the COVID-19 vaccine.

The Cronbach’s alpha coefficient and the range of scores of the model constructs, the mean and mean percentage of scores, and the Pearson correlation coefficient of the constructs of TRA regarding receiving the COVID-19 vaccine have been presented in Table 2. In this study, there was a positive and significant correlation between attitude (behavioral beliefs and outcomes evaluation) and subjective norms (normative beliefs and motivation to comply) with the COVID-19 vaccine receive intention.

**Table 2:** Bivariate Correlations of TRA Variables and COVID-19 vaccine receive intention

Variable	Item Number	α Cronbach’s	Range of score	Mean (SD)	mean percentage	1	2	3	4	5	6	7
1.Attitude	14	0.855	7–175	94.44(27.36)	53.96							
2.Behavioral Beliefs	**7**	0.811	7–35	24.73(4.64)	70.66	0.761^a^						
3.Outcome Evaluation	7	0.786	7–35	25.32(3.62)	72.34	0.420^a^	0.771^a^					
4.Subjective Norms	12	0.812	6–150	85.53(26.16)	57.02	0.732^a^	0.410^a^	0.702^a^				
5.Normative Beliefs	6	0.853	6–30	22.97(4.68)	76.56	0.669^a^	0.711^a^	0.404^a^	0.727^a^			
6.Motivation to Comply	6	0.801	6–30	21.77(3.67)	72.56	0.922^a^	0.643^a^	0.692^a^	0.824^a^	0.770^a^		
7.COVID-19 Vaccine Receive Intention	3	0.877	3–15	12.05(3.24)	80.33	0.948^a^	0.808^a^	0.623^a^	0.855^a^	0.461^a^	0.495^a^	

^a^ Correlation is significant at the 0.01 level

In order to determine the variables related to the COVID-19 vaccine receive intention, first the relationships of each of the independent variables (age, gender, occupation, education, marital status, chronic disease, smoking, place of residence, history of flu vaccine, history of COVID-19, information sources) with the behavioral intention variable were studied as univariate regression and variables with 0.25 ≥ *p*-value were entered into multivariate regression model (the enter method).

We set the p-value of 0.25 ≥ as the threshold for including variables in the multivariate model as suggested elsewhere as an appropriate threshold [18].

To test the collinearity of independent variables in the model, VIF was estimated. The estimated value was below 1.2 for all variables included within the regression.

The age group was divided into three categories that were made two dummy variables to enter the regression model, so that the age group of 18–29 years was considered as a reference and the age groups of 30–49 years and over 50 years were compared to the reference group, which showed that the age group did not predict vaccination.

There were also five categories of information sources that were made four dummy variables to enter the regression model, so that the group of friends and colleagues was considered as a reference and social media, radio and television, medical staff, scientific journals were compared to the reference group, which the results showed that the use of social media (β = 0.050, CI = 0.004, 0.648, *P* = 0.043) predicts the intention to receive COVID-19 vaccine. The findings summarized in Table 3 showed that Attitude (β = 0.497, CI = 0.055, 0.062, *P* < 0.001) and subjective norms (β = 0.394, CI = 0.045, 0.053, P < 0.001) also predicted the intention to receive COVID-19 vaccine.

**Table 3:** Multiple Regression Predicting Intention

Predictor	B	95.0% Confidence Interval for B		Beta	t	*p*-value
		Lower Bound	Upper Bound			
(Constant)	−4.728	−5.496	−3.959		−12.060	0.000
Age						
(18–29)	Reference					
(30–49)	0.077	−0.115	0.269	0.011	0.787	0.431
(50–75)	−0.001	−0.264	0.262	0.000	−0.010	0.992
Gender	0.008	−0.140	0.156	0.001	0.106	0.915
Marital status	−0.021	−0.200	0.157	−0.003	−0.236	0.814
Chronic diseases	0.109	−0.077	0.294	0.013	1.147	0.251
Smoking	−0.207	− 0.444	0.031	− 0.019	−1.705	0.088
Influenza vaccine	− 0.083	− 0.256	0.089	− 0.010	− 0.948	0.343
Source information						
Friend and colleague	Reference					
social media	0.322	0.004	0.648	0.050	2.934	0.043
Radio and television	0.278	−0.225	0.521	0.041	0.777	0.103
Treatment staff	0.148	−0.536	0.479	0.014	−0.110	0.437
scientific journals	−0.028	−0.056	0.612	−0.001	1.633	0.913
Attitude	0.059	0.055	0.062	0.497	31.938	0.000
Subjective Norms	0.049	0.045	0.053	0.394	25.411	0.000

Dependent Variable: COVID-19 Vaccine Receive Intention

R2 = 0.710

Adjusted R2 = 0.709

**Fig. 3:**
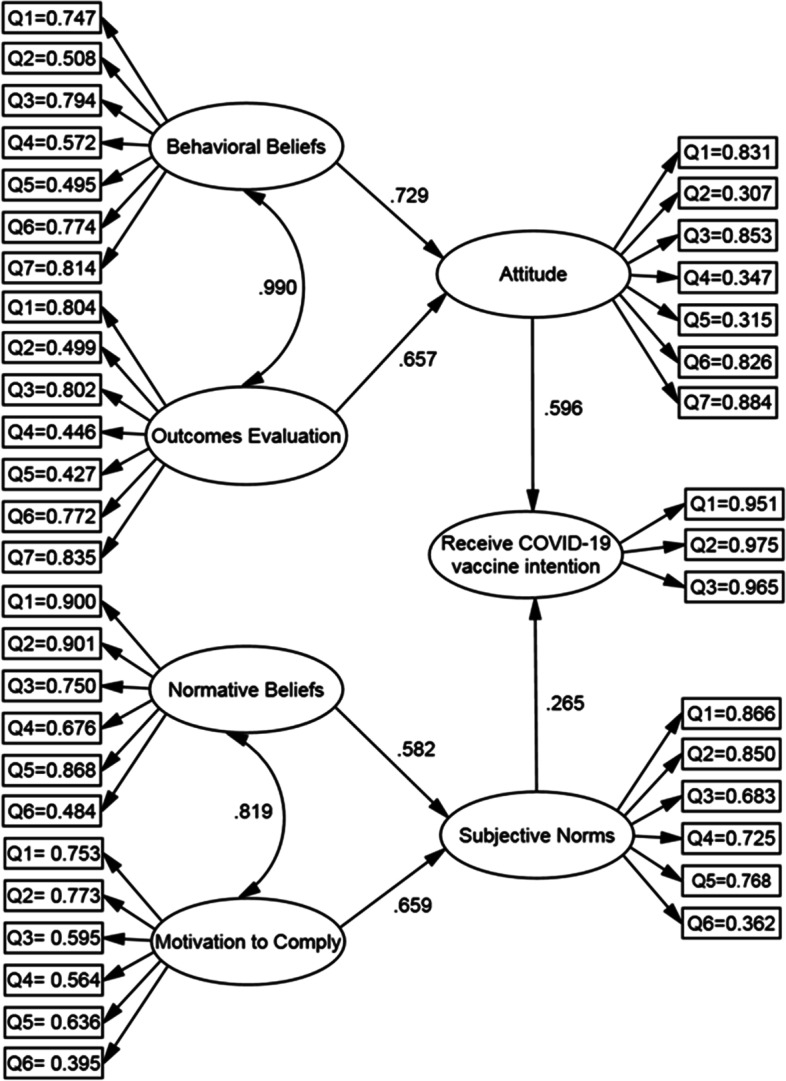
Structural equation modeling of COVID-19 vaccine ،*Significant at the 0.01 level

The final model of path analysis showed that the constructs of attitude (β = 0.596, P < 0.001), subjective norms (β = 0.265, P < 0.001) directly affect the COVID-19 vaccine receive intention and other constructs indirectly affect COVID-19 vaccine receive intention (Fig. 3).

In examining the direct, indirect and total effects, attitude with a total effect of 0.596 has the highest effect on the COVID-19 vaccine receive intention and behavioral beliefs, outcomes evaluation, subjective norms, motivation to comply and normative beliefs are in the next ranks, respectively (Table 4).

**Table 4:** Direct and indirect effects of model constructs on COVID-19 vaccine receive intention in path analysis

variable name	direct Effects	Indirect Effects	total Effects
Attitude	0.596	0.000	0.596
Subjective Norms	0.265	0.000	0.265
Behavioral Beliefs	0.000	0.434	0.434
Outcomes Evaluation	0.000	0.391	0.391
Normative Beliefs	0.000	0.154	0.154
Motivation to Comply	0.000	0.174	0.174

In general, the two attitude and subjective norms variables explain 64% of the variance of COVID-19 vaccine receive intention, the two behavioral beliefs and outcomes evaluation variables describe 37% of the attitude variance, and the two normative beliefs and motivation to comply variables describe 43% of the subjective norms variance (Table 5).

**Table 5:** Path coefficients and variance value explained by the constructs in the path analysis

path			β	S. E	C.R	p	R^2^
Attitude	→	COVID-19 Vaccine Receive Intention	0.596	0.003	36.272	<.001	0.640
Subjective Norms	→	COVID-19 Vaccine Receive Intention	0.265	0.002	19.118	<.001	
Behavioral Beliefs	→	Attitude	0.729	0.014	4.252	<.001	0.371
Outcomes Evaluation	→	Attitude	0.657	0.022	2.134	0.033	
Normative Beliefs	→	Subjective Norms	0.582	0.794	9.331	<.001	0.438
Motivation to Comply	→	Subjective Norms	0.659	0.904	14.449	<.001	

Note. *SE* Standard Error; *CR* Composite Reliability

According to the results of Table 6, the fit indices to evaluate the totality of the final model of path analysis show that in general the model has a very good fitness.

**Table 6:** Model fit index of predictive pattern of COVID-19 vaccine receive intention rate in path analysis

Χ^**2**^	DF	Χ^**2**^ / DF	GFI	AGFI	NFI	CFI	IFI	RMSEA	RMR
4801	907	5.293	0.906	0.898	0.927	0.916	0.885	0.036	0.255

Goodness-of-Fit Statistic (GFI); Adjusted Goodness-of-Fit Statistic (AGFI); Normed-fit index (NFI); Comparative fit index (CFI); Incremental Fit Indices (IFI); Root Mean Square Error of Approximation (RMSEA); Root Mean Square Residual (RMR)

## Discussion

This study was aimed to predict the COVID-19 vaccine receive intention based on the TRA in the population over 18 years of age in the South of Iran. Findings of the study showed that attitude, subjective norms and the use of social media predicted the COVID-19 vaccine receive intention, which the attitude had more predictive power.

In this study, 78% of people intended to receive the vaccine if it was their turn to receive the COVID-19 vaccine. A study conducted in *Hong Kong* showed vaccination intention is equal to 44.2% and an online survey in China showed it is equal to 54.6% [19, 20]. The rate of COVID-19 vaccination intention is reported 65.7% in Japan [21], 53.1% in Kuwait [22], 64% in UK [23] 78.3% in Indonesia [24] and 64.7% in Saudi Arabia [25]. A survey in Europe found that on average 73.9% of respondents from Germany, the England, Denmark, the Netherlands, France, Portugal and Italy were willing to receive the COVID-19 vaccine [26]. An online survey in the United States showed that 69% of participants intended to be vaccinated against COVID-19 [13]. One of the reasons for the high intention of people to be vaccinated in Iran compared to some other countries, can be attributed to the outbreak and mortality caused by this disease simultaneously with the fifth peak of the disease in Iran and the outbreak of virus new variant such as Delta at the time of the conducting study.

Studies have shown that fear of COVID-19 and the possibility of infected with COVID are effective in justifying people’s intention to vaccinate COVID-19 [10]. Also, the severity of COVID-19, the vaccine receptors’ self-efficacy, and the effectiveness of vaccine in preventing infection were effective in the Iranian population’s intention to receive COVID-19 vaccine [27].

In the present study, the mean score of intention and subjective norms in the age group over 50 years was significantly higher than other age groups, which is consistent with other studies [28, 29]. Studies in Japan and America have shown that elderly people were more likely to receive COVID-19 vaccination than younger people [21, 30]. These findings are also consistent with other studies in the United States, England and Ireland [31, 32]. To justify this issue, we can say that the risk of COVID-19 and mortality caused by COVID-19 increase with age [33], and people over the age of 50 may have underlying disease and mortality caused by COVID-19 is more common in these individuals than other groups [34]. It is suggested to plan for informing people under 50 about the benefits of vaccination through appropriate educational content and communication channels. To increase the rate of vaccination, advertising and educational campaigns on the safety and efficacy of COVID-19 vaccines lead to increase public confidence to these vaccines and increase vaccination [35–37]. Studies have shown that public concerns about safety and side effects of the vaccine are among the key variables influencing vaccination decisions, especially for newly developed vaccines [38–40]. Trust in the healthcare system was positively associated with willingness to get COVID-19 vaccination and generalized trust was positively associated with willingness to get COVID-19 vaccination [41].

The findings of the present study showed that the mean score of attitude, subjective norms and COVID-19 vaccine receive intention in people with chronic disease is significantly higher than other people, which regarding that the presence of chronic disease causes severe involvement and death in people with COVID-19 [34], it is not unexpected and is consistent with other studies [29]. However, this matter should not cause that other people without chronic disease, do not consider themselves at risk and do not have COVID-19 vaccine receive intention, because receiving vaccine by 70–80% of the society people will be effective and efficient [42].

The findings of this study also showed that the mean score of attitude, subjective norms and intention in people who do not smoke is significantly higher than smoke people. Since smoking is one of the most known important risk factors for respiratory infections, it can make a person vulnerable against the coronavirus and lead to more severe illness, if they become infected [43]. It is recommended that the necessary interventions should be designed to inform and alter attitudes and ultimately COVID-19 vaccine receive intention in these individuals, and a multilateral approach should be used to fight distrust towards the COVID-19 vaccine in this high-risk group.

Findings of the study showed that the mean score of attitude and intention in people who had a history of receiving flu vaccine is significantly higher than other people, some studies have shown that receiving the flu vaccine last year is a strong predictor of the tendency to receive the COVID-19 vaccine. It seems that a positive attitude towards COVID-19 vaccine is associated with a positive opinions towards receiving other vaccines [44].

Our study showed that attitude is the strongest predictor of COVID-19 vaccine receive intention, which was consistent with other studies [45]. Attitude construct was influenced by the construct of behavioral beliefs and outcomes evaluation, which behavioral beliefs had a greater effect on attitudes than outcomes evaluation; belief in the effectiveness of the Covid − 19 vaccine significantly influenced behavioral beliefs that are consistent with other studies [2, 46]. Other studies have shown that a positive attitude towards vaccination is associated with factors such as reducing the risk of infection, improving socioeconomic recovery and returning to a normal life, and in contrast, a negative attitude was associated with underestimating the severity of the disease, low effectiveness of the Covid vaccine and more belief in the natural immune system and distrust to the government or vaccines, and a lack of accurate vaccine news [47, 48]. Positive attitudes towards vaccination can be created through media support and the strategic use of social media [44].

The findings of our study showed that subjective norms also predict the COVID-19 vaccine receive intention. This means that those who are positive about social pressure and have a positive normative belief, have more COVID-19 vaccine receive intention. The construct of subjective norms was influenced by the constructs of normative beliefs and motivation to comply, which the motivation to comply had a greater effect on subjective norms than normative beliefs. In this study, the family opinion to receive COVID-19 vaccine had the most important and effective role, which effective interventions should be planned to train families and encourage family members to receive the vaccine in various ways and based on the culture of the region. Also in this study, the opinion of friends, colleagues and doctors was positively related to COVID-19 vaccine receive intention, which is consistent with other studies [29, 44, 45, 49]. A major reason is probably that neighbors, family and friends have a strong and lasting effect on people’s minds in Iranian society [11]. There are also many family and friendly relationships in the southern provinces of Iran, and this culture affects collective measures and norms during the COVID-19 pandemic [50]. Therefore, the behavior of the general public is also important in order to change the behavior of other people in the community, and to achieve this, representatives of the community should be used in planning, implementation and evaluation of relevant programs, as well as identifying and introducing credible and official sources. Regarding the importance of physicians’ advice to receive vaccination in this study and other studies, the justification of physicians and healthcare professionals to encourage people conducting vaccination has particular importance [44, 51].

Planning to motivate and encourage people to share their positive thoughts and experiences about receiving the COVID-19 vaccine through social media and cyberspaces can be effective in increasing vaccine acceptance. It is also recommended to use popular and influential people in the community such as leaders, athletes and artists or physicians and health professionals to promote vaccination and influence people in the community.

The results of this study showed that attitudes play a greater role in the COVID-19 vaccine receive intention than subjective norms, which is consistent with other studies [52], which can be because this study was conducted online and most participants in the study had bachelor degree and above, and attitude is often more important and decisive than subjective norms in the upper socioeconomic classes and people with higher literacy level.

The findings of our study showed that the use of social media predicts the COVID-19 vaccine receive intention and people whose their source of information about the vaccine is social media, have significantly higher COVID-19 vaccine receive intention than others. In this regard, it can be said that it is necessary to increase the level of health literacy and media literacy of individuals and monitor the content produced in social media, and identifying rumors and informing people about this issue should also be planned. Because negative information, spread through social media, about COVID-19 vaccines was associated with a lower acceptability among the community [53]. Besides, much information is available in different channels of social media about the safety and efficacy of vaccines. This information can be largely inaccurate, misleading and biased. It can increase people’s suspicion towards vaccination [44]. Therefore, providing correct information through guided information and educational campaigns and credible media is essential to reassure people who are skeptical as a result of false and fake information about receiving vaccine [54].

### Study limitations

First, because we selected participants to study through an online survey platform, those who do not have access to online surveys (e.g., the elderly, rural), people who do not have access to the Internet and people with low literacy, entered the study less than others. Second, due to the fact that at the time of the study, vaccination in Iran was performed only for people over 75 years of age and the medical staff, the response of individuals may be different if there is a vaccine.

### Study strengths

- Use a strong, fully tested and complete theory to explain the intention of the people and the factors influencing it
- Data collection from 4 southern provinces of Iran in rural and urban areas
- Synchronization time coincides with pandemic peak time

## Conclusions

The present study showed that the TRA could be a good predictor COVID-19 vaccine receive intention, so that attitudes and subjective norms significantly predicted COVID-19 vaccine receive intention. The use of social media as a source of information can also be a predictor of receiving vaccine. For this reason, comprehensive training programs about receiving vaccine are recommended to reinforce attitudes and subjective norms about receiving vaccine. Therefore, it can be said that we can strengthen the attitudes and subjective norms of individuals and increase the intention of people in the community to receive the COVID-19 vaccine by providing appropriate and credible platforms to implement virtual information and education programs and conducting positive interventions.

In particular, informing and training families is very important to persuade their members. Also, positive vaccine advertisements that show that celebrities and important officials have been vaccinated, may increase subjective norms and consequently improve the willing to vaccinate COVID-19 among the general public. In addition, it is suggested that the reasons of not receiving the COVID-19 vaccine in people who do not intend to do it, should be investigated in order to design effective interventions.

## Supplementary Information

**Additional file 1.** Questionnaire.

## Data Availability

The datasets used and/or analyzed during the current study are available from the corresponding author on reasonable request.

## Abbreviations

TRA: The Theory of Reasoned Action
SARS: Severe Acute Respiratory Syndrome
COVID19: Corona Virus Disease 2019
