# Supplementary material for: Predicting the COVID-19 vaccine receive intention based on the theory of reasoned action in the south of Iran
Source: BMC Public Health. 2022 Feb 4;22:229. doi: 10.1186/s12889-022-12517-1 (PMC8814221; doi:10.1186/s12889-022-12517-1)
Supplement: Supplementary file 1 — Additional file 1. Questionnaire. [file 12889_2022_12517_MOESM1_ESM.docx]

**Dear compatriot**

**Hi**

This questionnaire was designed by the Faculty of Health of *Hormozgan* University of Medical Sciences in order to predict the **COVID-19 vaccine receive intention based on the theory of reasoned action in Iran**. Please read it carefully and answer its questions. You also do not need to write your first name and last name. If you have any ambiguities in answering the questions, please call *Ms. Ezzati* at 09357615901.

**Attention:**

1. In answering questions, your personal opinions are important to us and there is no need to consult with others.

2. People who have not yet received the Covid-19 vaccine are asked to answer these questions.

3. Please answer this questionnaire for people 18 years and older.

**Personal details:**

Age: ........ years

Gender: 1. Male 2. Female

Marital status: 1. Single 2. Married 3. Divorced 4. Widow

Education level:

1. Elementary 2. Secondary 3. Diploma 4. Associate Degree 5. Bachelor and higher

Occupation: 1- Worker □ 2- Public sector employee □ 3- Private sector employee □ 4- Teacher □ 5- Businessman □ 6- Specialized jobs (doctor and engineer) □ 7- Housewife □ 8- Student □ 9-self-employed □ 10- Unemployed □ 11- Medical staff □ 12-Retired □ 13- Other cases □

Have you ever received the Covid-19 vaccine? 1. Yes 2. No

Do you have a history of the following diseases?

Blood pressure: 1. Yes 2. No

Diabetes: 1. Yes 2. No

Kidney Diseases: 1. Yes 2. No

Cardiovascular diseases: 1. Yes 2. No

Do you smoke?

1. Yes 2. No

Place of resident: 1. City 2. Village

Province of residence:

Have you ever received the flu shot? 1. Yes 2. No

Have you ever infected with Covid-19 disease? Yes No

If you have Covid-19 disease, is your PCR test positive? Yes No

Has anyone in your family ever had Covid-19 disease? Yes No

What do you know most about Covid-19 vaccines?

1- Radio and TV 2- Social media (Instagram, Twitter and WhatsApp, others) 3- Healthcare staff 4- Scientific journals 5- Friends and colleagues

**Attitude:**

Behavioral beliefs

1. If I receive the Covid-19 vaccine, I will be protected from Covid-19.

1- Strongly agree 2- Agree 3- I have no idea 4- Disagree 5- Strongly disagree

**2. If I receive the Covid-19 vaccine, I will suffer from side effects from the vaccine**

1- Strongly agree 2- Agree 3- I have no idea 4- Disagree 5- Strongly disagree

3. I believe in the efficacy and safety of the available Covid-19 vaccines.

1- Strongly agree 2- Agree 3- I have no idea 4- Disagree 5- Strongly disagree

4. If I receive the Covid-19 vaccine, I still have to observe the health protocols.

1- Strongly agree 2- Agree 3- I have no idea 4- Disagree 5- Strongly disagree

**5. Thinking about receiving the Covid-19 vaccine worries me.**

1- Strongly agree 2- Agree 3- I have no idea 4- Disagree 5- Strongly disagree

6. If I receive the Covid-19 vaccine, I will be mentally and emotionally relieved.

1- Strongly agree 2- Agree 3- I have no idea 4- Disagree 5- Strongly disagree

7. Covid-19 vaccination is an effective method to prevent and control this disease.

1- Strongly agree 2- Agree 3- I have no idea 4- Disagree 5- Strongly disagree

Outcome’s evaluation:

1- Protection against Covid-19 disease

1- Strongly desirable 2- desirable 3- I have no opinion 4- Undesirable 5- Strongly undesirable

**2 - Side effects due to receiving the vaccine**

1- Strongly desirable 2- desirable 3- I have no opinion 4- Undesirable 5- Strongly undesirable

3- Efficacy and safety of Covid-19 vaccines

1- Strongly desirable 2- desirable 3- I have no opinion 4- Undesirable 5- Strongly undesirable

4 – observing the health protocols even if receiving the Covid-19 vaccines

1- Strongly desirable 2- desirable 3- I have no opinion 4- Undesirable 5- Strongly undesirable

5 - Concerns about receiving the Covid-19 vaccines

1- Strongly desirable 2- desirable 3- I have no opinion 4- Undesirable 5- Strongly undesirable

6 - Mentally and emotionally relief after receiving the Covid-19 vaccines

1- Strongly desirable 2- desirable 3- I have no opinion 4- Undesirable 5- Strongly undesirable

7 - Prevention and control of Covid-19 vaccines

1- Strongly desirable 2- desirable 3- I have no opinion 4- Undesirable 5- Strongly undesirable

**Subjective norms**

Normative beliefs

1. My family members recommend that I receive the Covid-19 vaccine if it is my turn to be vaccinated.

1- Strongly agree 2- Agree 3- I have no idea 4- Disagree 5- Strongly disagree

2. My family members agree to receive the Covid-19 vaccine.

1- Strongly agree 2- Agree 3- I have no idea 4- Disagree 5- Strongly disagree

3 - My colleagues and friends encourage me to receive the Covid-19 vaccine.

1- Strongly agree 2- Agree 3- I have no idea 4- Disagree 5- Strongly disagree

4 - Doctors pursue me to receive the Covid-19 vaccine.

1- Strongly agree 2- Agree 3- I have no idea 4- Disagree 5- Strongly disagree

5 - Most people who are important for me recommend to receive the Covid-19 vaccine.

1- Strongly agree 2- Agree 3- I have no idea 4- Disagree 5- Strongly disagree

6 - Cyberspaces (WhatsApp, Instagram, etc.) prohibit me to receive the Covid-19 vaccine.

1- Strongly agree 2- Agree 3- I have no idea 4- Disagree 5- Strongly disagree

Motivation to comply

1. Family members' advice to receive the Covid-19 vaccine is ……. for me.

Very important □ important□ I have no idea□ not important□ never important□

2- My family agreement to receive the Covid-19 vaccine is……. for me.

Very important □ important□ I have no idea□ not important□ never important□

3- The opinion of my friends and colleagues to receive the Covid-19 vaccine is ……. for me.

Very important □ important□ I have no idea□ not important□ never important□

4- Doctors' opinion to receive Covid-19 vaccine is ……. for me.

Very important □ important□ I have no idea□ not important□ never important□

5- The opinion of important people to receive the Covid-19 vaccine is ……. for me.

Very important □ important□ I have no idea□ not important□ never important□

**6- The opinion of cyberspaces about not receiving the Covid-19 vaccine is ……. for me.**

Very important □ important□ I have no idea□ not important□ never important□

**COVID-19 vaccine receive intention**

1- I intend to receive the vaccine if it is my turn to receive Covid-19 vaccine.

1- Strongly agree 2- Agree 3- I have no idea 4- Disagree 5- Strongly disagree

2- I have planned to receive this vaccine if it is my turn.

1- Strongly agree 2- Agree 3- I have no idea 4- Disagree 5- Strongly disagree

3- My opinion and wish are to receive the Covid-19 vaccine.

1- Strongly agree 2- Agree 3- I have no idea 4- Disagree 5- Strongly disagree
